# PB2 segment promotes high-pathogenicity of H5N1 avian influenza viruses in mice

**DOI:** 10.3389/fmicb.2015.00073

**Published:** 2015-02-10

**Authors:** Hailiang Sun, Pengfei Cui, Yafen Song, Yan Qi, Xiaokang Li, Wenbao Qi, Chenggang Xu, Peirong Jiao, Ming Liao

**Affiliations:** College of Veterinary Medicine, South China Agricultural UniversityGuangzhou, China

**Keywords:** influenza virus, H5N1, virulence, reassortant, mutants, mice

## Abstract

H5N1 influenza viruses with high lethality are a continuing threat to humans and poultry. Recently, H5N1 high-pathogenicity avian influenza virus (HPAIV) has been shown to transmit through aerosols between ferrets in lab experiments by acquiring some mutation. This is another deeply aggravated threat of H5N1 HPAIV to humans. To further explore the molecular determinant of H5N1 HPAIV virulence in a mammalian model, we compared the virulence of A/Duck/Guangdong/212/2004 (DK212) and A/Quail/Guangdong/90/2004 (QL90). Though they were genetically similar, they had different pathogenicity in mice, as well as their 16 reassortants. The results indicated that a swap of the PB2 gene could dramatically decrease the virulence of rgDK212 in mice (1896-fold) but increase the virulence of rgQL90 in mice (60-fold). Furthermore, the polymerase activity assays showed that swapping PB2 genes between these two viruses significantly changed the activity of polymerase complexes in 293T cells. The mutation Ser715Asn in PB2 sharply attenuated the virulence of rgDK212 in mice (2710-fold). PB2 segment promotes high-pathogenicity of H5N1 avian influenza viruses in mice and 715 Ser in PB2 plays an important role in determining high virulence of DK212 in mice.

## Introduction

Recent research has confirmed that obtaining human receptor binding specificity and enhancing the polymerase activity enabled the H5N1 virus to achieve aerosol transmission between ferrets (Herfst et al., 2012; Imai et al., 2012). This may allow novel H5N1 virus to become the next pandemic. H5N1 HPAIV is a continued threat to public health (Chen et al., 2004, 2005, 2006; Li et al., 2010; Wan et al., 2011). Therefore, it is pertinent to continue studying the pathogenesis mechanism of H5N1 HPAIV.

Several genes are confirmed as contributing to the virulence and replication of H5N1 viruses. The PB2 and NS genes play an important role in determining the pathogenicity of the influenza virus to a host. Specific adaptation of polymerases was important in determining viral host range and virulence (Hatta et al., 2007; Neumann et al., 2007). Mutations occurring at key positions in PB2, such as 158, 271, 627, and 701, could significantly enhance the replication of H5N1 influenza A virus in mammalian cells as well as pathogenicity and transmission in animal models (Hatta et al., 2001; Li et al., 2005; Gao et al., 2009; Bussey et al., 2010; Zhou et al., 2011). The virus' capability to inhibit α/β-interferon production obviously affected its level of virulence to its host. The length of NS1 protein or mutation at position 92/42 affected the virulence of viruses in mice and pigs by inhibiting production of host interferon (Seo et al., 2002; Quinlivan et al., 2005; Jiao et al., 2008). The PB1-F2 Asn66Ser variant also reduced the production of host IFN α/β (Conenello et al., 2007). In addition, mutations at receptor binding sites and disappearance of N-glycosylation motif in HA protein affected the binding feature and virulence (Hatta et al., 2001; Maines et al., 2006; Chen et al., 2007; Tumpey et al., 2007; Yen et al., 2007a; Gao et al., 2009; de Wit et al., 2010). Mutations in the M1 and NA genes could possibly promote viral replication in mammalian cells and enhance the lethality of the virus in mice (Yen et al., 2007b; Fan et al., 2009; van Wielink et al., 2012).

However, the mammalian pathogenic mechanism of H5N1 HPAI virus is still not clear. In this study, we explored the molecular determinants of different virulence in mice between DK212 and QL90, by engineering single segment swap reassortant viruses and single amino acid mutation viruses using reverse genetics technology.

## Materials and methods

### Virus, sequence, and mouse

DK212 and QL90 and high-pathogenicity H5N1 influenza viruses were isolated from ducks/quails in China, purified and propagated in 9- to 11-day-old specific-pathogen-free (SPF) embryonic hen eggs, and stored at −80°C. Viral RNA was extracted from allantoic fluid with the RNeasy Mini kit (Qiagen) and reverse transcribed with Superscript III (Invitrogen) by using the primer of 12 units. PCR amplification was performed using a set of special primers (Hoffmann et al., 2001). PCR products were purified using the PCR Purification Kit (Promega) and sequenced by Shanghai Invitrogen Biotechnology Co., Ltd. Sequenced data were compiled with the SEQMAN program of Lasergene7 (DNASTAR). According to the characteristics of the HA antigen, DK212 belonged to Clade 9 (Sun et al., 2011), and QL90 was in Clade 1. The two viruses shared the same NA and NP genes but displayed 36 differences at the amino acid level in other six genes (Table 1). Six-week-old, female SPF BALB/c mice were purchased from the Laboratory Animal Center of South China in Guangzhou.

**Table 1:** **Amino acid differences between DK212 and QL90**.

**Gene**	**Difference and position of amino acids**							
PB2	Gln^a^39^b^Lys^c^	Thr339Lys	Arg340Lys	Gly368Arg	Ile649Val	Thr684Ala	Ser715Asn	–^d^
PB1	Arg353Lys	–	–	–	–	–	–	–
PA	Leu261Phe	Asn296Ser	Glu327Gly	Thr337Ala	Val354Ile	Lys544Glu	Asn648Ser	Ser653Pro
HA	Ser100Asn	Ala143Thr	Leu154Gln	Pro157Ser	Thr172Ala	Lys205Arg	Asn289Ser	–
NP	–	–	–	–	–	–	–	–
NA	–	–	–	–	–	–	–	–
M1	Met59Ile	–	–	–	–	–	–	–
M2	Pro25Leu	Ser31Asn	Glu66Ala	Asn82Ser	–	–	–	–
NS1	Val129Phe	Tyr133Phe	Asp166Gly	Asn201Ser	–	–	–	–
NS2	Met14Val	Met49Val	Ile83Val	Thr115Ala	–	–	–	–

a *The amino acid at corresponding position of DK212*.

b *The position at which amino acids of the two viruses is different*.

c *The amino acid at corresponding position of QL90*.

d *The protein sequence of DK212 is same to that of QL90*.

### Generation of viruses

The cDNA of genes from DK212 or QL90 were amplified by a set of primers (Supplementary Tables 1, 2), and inserted into a pHH vector using ESP3I enzyme cutting sites. Single mutations were introduced into the PB2 gene by QuikChange II XL Site-Directed Mutagenesis (Agilent Technologies) with a set of primers (Supplementary Table 3). Viruses were rescued by using an eight-plasmid reverse genetics system (Hoffmann et al., 2000). The viruses obtained from cloned DK212 or QL90 cDNA were designated rgDK212 and rgQL90, respectively. The viruses bearing the PB2, PB1, PA, HA, NA, NP, M, or NS gene of QL90 and the other seven genes of DK212 were designated 212-90PB2, 212-90PB1, 212-90PA, 212-90HA, 212-90NP, 212-90NA, 212-90M, and 212-90NS, respectively. The viruses bearing the PB2, PB1, PA, HA, NA, NP, M, or NS gene from DK212 and the remaining genes from QL90 were designated 90-212PB2, 90-212PB1, 90-212PA, 90-212HA, 90-212NP, 90-212NA, 90-212M, and 90-212NS, respectively. Mutant DK212 viruses containing a substitution of the PB2 amino acid residue at position Gln39Lys, Ile649Val, Thr684Ala, and Ser715Asn were designated 212-Gln39Lys, 212-Ile649Val, 212-Thr684Ala, and 212-Ser715Asn. Likewise, mutant QL90 viruses were designated 90-Lys39Gln, 90-Val649Ile, 90-Ala684Thr, and 90-Asn715Ser, respectively. Rescued viruses were then sequenced to conclude that there was no unwanted mutation.

### Pathogenicity of viruses to mice

To evaluate the pathogenicity of these viruses in mice, 6-week-old, female SPF BALB/c mice were randomly divided into groups of 8 mice. After being lightly anesthetized with CO_2_, the mice were intranasally inoculated with the corresponding viruses, at a dose of 10^6^ EID_50_ in a 0.05 mL volume. Additionally, five mice inoculated with 0.05 mL PBS served as negative controls. Three mice in each group were euthanized at 3 days post-inoculation (DPI) to determine virus titers in their brain, spleen, kidneys and lungs, as previously described (Chen et al., 2004). Virus titers were calculated as means ± standard deviation in log_10_EID_50_/gram of tissue by using SPASS (Version 11.5), and the data were analyzed using a One-Way repeated-measure analysis of variance (ANOVA) followed by Turkey and Duncan's multiple comparison test. The remaining mice were investigated daily for 14 days to observe weight loss and mortality. Variation of body weight was detected according to previous study (Li et al., 2005; Jiao et al., 2008; Fan et al., 2009). Briefly, eight mice in group were weighed and then the total weight was divided by eight to get mean weight during 1–3 post infection days (DPI). After 3 DPI, total alive mice in the group were weighed and then divided the number of live mice to get meant weight. The mean body weight at time point minus the mean body weight at 0 DPI and then the result was divided the original mean body weight go get the percentage of weight loss. To detect the 50% mouse lethal dose (MLD_50_) of viruses, seven groups of five mice each were infected with 10-fold serial dilutions, from 1 EID_50_ to 10^6^ EID^16^_50_. These mice were daily investigated and weighed for 14 days. Mice which lost more than 30% of their body weight were euthanized. MLD_50_ were calculated using the Reed-Muench method (Reed and Muench, 1938).

### Polymerase activity assay

To detect the polymerase activity of viruses, the plasmid pMD18T-NP-LUCI was constructed. In a brief explanation, the luciferase gene flanked with the non-coding region of NP of DK212 was amplified, using pGL-3 control (Promega) as template, by primer L-NPFL-NPR. It was then cloned into pMD18-T. 293T cell was cultured in a 12-well plate overnight. When the monolayer formed approximate 90%, they were transfected with 0.4μg of pHH-NP, pHH-PA, pHH-PB1, and pHH-PB2, and 0.1μg of pMD-18T-NP-LUCI, as well as 0.1μg pRL-SV40 (Promega) as an internal control. After transfection for 24h, cell lysate was prepared and the luciferase yield was tested by using the dual-luciferase reporter assay system (Promega), with a single tube luminometer (Promega). Relative activity of the polymerase was normalized to Renilla gene expression. The results were calculated as means ± standard deviations from three independent experiments, using SPASS (Version 11.5).

### Biosafety and animal handling

The laboratory and mice experiments were carried out under BSL-3 conditions in compliance with biosafety committee of South China Agriculture University approved protocols. The handling of mice was performed in accordance with experimental animal administration and ethics committee of South China Agriculture University approved guideline.

## Results

### The virulence of DK212 was stronger than QL90 to BABL/c mouse

**Figure 1:**
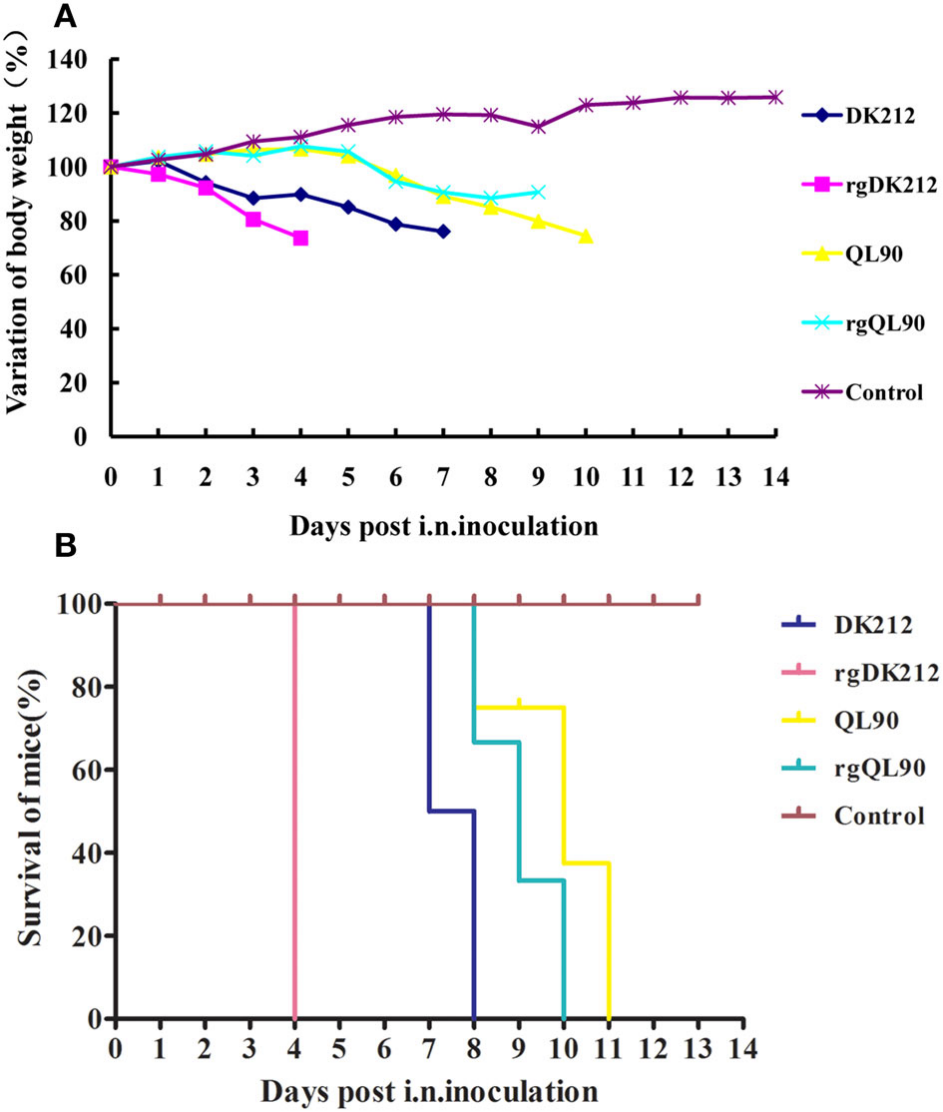
**Lethality and weight variation of BABL/c mice caused by DK212, QL90, rgDK212, and rgQL90**. **(A)** Weight variation of BABL/c mouse during the 14 days post-inoculation. The mean weight variation of the mice infected with DK212, QL90, rgDK212, and rgQL90 at the doses of 10^6^ EID_50_. **(B)** Death patterns of the mice infected with DK212, QL90, rgDK212, and rgQL90 with the doses of 10^6^ EID_50_.

To compare the pathogenicity of DK212 and QL90, mice were intranasally infected with the corresponding virus at a dose of 10^6^ embryo infective dose 50 (EID_50_). DK212 quickly caused 23.97% body weight loss and killed all mice by 7 DPI at a dose of 10^6^ EID_50_. Virus was isolated from the brain, spleen, kidney, and lung, with a mean titer between 1.67 and 6.25 log_10_EID_50_ on 3 DPI. Furthermore, the virus titer of DK212 was higher than that of QL90 in the brain and kidney (*p* < 0.05). Although QL90 caused 25.54% body weight loss and killed all the mice at 10 DPI in a dose of 10^6^ EID_50_, the virus only replicated in the spleen and lungs with a mean titer between 3 and 5.83 log_10_EID_50_ (Figures 1A,B and Table 2). The MLD_50_ of DK212 was 1.5 log_10_EID_50_, while the MLD_50_ of QL90 was 4.167 log_10_EID_50_ (Table 3). The virulence of DK212 in mice was 464-fold higher than that of QL90.

**Table 2:** **Virus titers in organs of BABL/c mice at 3 days post-intranasal inoculation of viruses**.

**Viruses**	**3 days post i.n. inoculation (log_10_ EID_50_/tissue/0.1 mL) ± SD**			
	**Brain**	**Spleen**	**Kidney**	**Lung**
DK212	2.58 ± 0.29^a^	1.67 ± 1.15^a^	2.92 ± 0.29^a^	6.25 ± 0
rgDK212	2.33 ± 0.63	3.5 ± 0.66	2.25 ± 0.5	6.58 ± 0.52
212-90PB2	<^b^	<^b^	<^b^	4.75 ± 0.5<^b^
212-90PB1	1.58 ± 1.01	2.92 ± 0.76	2.75 ± 0.43	6.42 ± 0.76
212-90PA	1.83 ± 0.88	4.08 ± 0.29	2.42 ± 0.76	5.67 ± 0.38
212-90HA	1.25 ± 0.43	1.92 ± 0.58^b^	<^b^	6.58 ± 0.29
212-90NP	1.92 ± 1.15	3.17 ± 0.14	2.75 ± 0.5	5.58 ± 1.26
212-90NA	1.25 ± 0.43	2.42 ± 1.04	2.83 ± 0.52	5.75 ± 0.43
212-90M	<^b^	1.08 ± 0.14^b^	1.42 ± 0.72^b^	5.42 ± 0.29^b^
212-90NS	2.92 ± 0.29	3 ± 0.75	3.17 ± 0.14^c^	6.75 ± 0
212-Gln39Lys	1.25 ± 0.43	3.83 ± 0.14	3.08 ± 0.14^d^	7 ± 0
212-Ile649Val	2.17 ± 1.13	2.75 ± 1.39	2.17 ± 0.14	6.92 ± 0.14
212-Thr684Ala	1.75 ± 0.66	2.67 ± 1.18	1.08 ± 0.14^e^	6.92 ± 0.14
212-Ser715Asn	1.33 ± 0.58	3.25 ± 1.9	2.08 ± 0.76	6.67 ± 0.38
QL90	<	3 ± 1.52	<	5.83 ± 0.52
rgQL90	<	2.5 ± 1.39	<	5.25 ± 0.43
90-212PB2	<	2.75 ± 0.87	<	5.25 ± 0.43
90-212PB1	<	<^f^	<	4.08 ± 0.14^f^
90-212PA	<	2.83 ± 1.59	1.92 ± 1.59	5.25 ± 0.5
90-212HA	1.42 ± 0.72	3.67 ± 0.38	2.33 ± 0.95^g^	6.17 ± 0.14^g^
90-212NP	<	2.17 ± 1.01	<	5.08 ± 0.14
90-212NA	<	3.75 ± 0.43	1.92 ± 0.29	5.75 ± 0.5
90-212M	1.08 ± 0.14	2.92 ± 1.01	1.75 ± 0.5	5.42 ± 0.29^g^
90-212NS	1.83 ± 0.88^g^	3.08 ± 0.76	1.58 ± 1.01	6.17 ± 0.52^g^
90-Lys39Gln	<	<^h^	<	5.08 ± 1.04
90-Val649Ile	<	<^h^	<	5.75 ± 0.5
90-Ala684Thr	<	1.33 ± 0.58	1.08 ± 0.14	4.92 ± 0.14
90-Asn715Ser	<	<^h^	<	3.58 ± 0.58^h^

*“<” means no virus was detected from the undiluted sample in three embryonated hen eggs. Virus titers are expressed as means ± standard deviation in log_10_EID_50_/tissue/0.1 mL*.

a *The titer of DK212 is higher than that of QL90(P < 0.05)*.

b *The titer of chimeric viruses is lower than that of rgDK212 (P < 0.05)*.

c *The titer of chimeric viruses is higher than that of rgDK212 (P < 0.05)*.

d *The titer of mutants is higher than that of rgDK212 (P < 0.05)*.

e *The titer of mutants is lower than that of rgDK212 (P < 0.05)*.

f *The titer of chimeric viruses is lower than that of rgQL90 (P < 0.05)*.

g *The titer of chimeric viruses is higher than that of rgQL90 (P < 0.05)*.

h *The titer of mutants is lower than that of rgQL90 (P < 0.05)*.

**Table 3:** **The MLD_50_ of the wild-type viruses and their chimeric viruses as well as mutants**.

**Viruses**	**MLD_50_ (log_10_ EID_50_)**	**Viruses**	**MLD_50_ (log_10_ EID_50_)**
DK212	1.5	QL90	4.167
rgDK212	1.167	rgQL90	4.17
212-90PB2	4.445	90-212PB2	2.4
212-90PB1	1.167	90-212PB1	3.445
212-90PA	2.045	90-212PA	6.25
212-90HA	0.375	90-212HA	4.167
212-90NP	1.375	90-212NP	4.445
212-90NA	2.167	90-212NA	4.167
212-90M	2.375	90-212M	4.276
212-90NS	1.167	90-212NS	2.045
212-Gln39Lys	3.6	90-Lys39 Gln	4.75
212-Ile649Val	2.464	90-Val649 Ile	5.25
212-Thr684Ala	3.75	90-Ala684Thr	5
212-Ser715Asn	4.6	90-Asn715Ser	4.625

*The 50% mouse lethal dose (MLD_50_) was evaluated by infecting groups of five mice with 10-fold serial dilutions, from 1 EID_50_ to 10^6^ EID_50_. Mice were investigated dailyand weighed for 14 days. Mice that lost body weight over 30% were euthanized. MLD_50_ were calculated with Reed-Muench method*.

The pathogenicity of the rescued viruses was similar to that of the wild-types. rgDK212 caused 26.36% body weight loss and killed all the mice at 4 DPI at a dose of 10^6^ EID_50_, and replicated in four organs with a titer of 2.25–6.58 log_10_EID_50_. Meanwhile, rgQL90 caused 10.36% body weight loss and caused 100% of the mice to die by 9 DPI at a dose of 10^6^ EID_50_ (Figures 1A,B). rgQL90 only replicated in the spleen and lungs with a mean titer of 2.5 log_10_EID_50_ and 5.25 log_10_EID_50_, respectively (Table 2). The MLD_50_ of rgDK212 was 1.17 log_10_EID_50_ and the MLD_50_ of rgQL90 was 4.17 log_10_EID_50_ (Table 3). rgDK212 and rgQL90 exhibited similar properties to their original viruses in terms of viral replication and MLD_50_.

### The swap of PB2 could dramatically decrease the virulence of DK212 in mice

**Figure 2:**
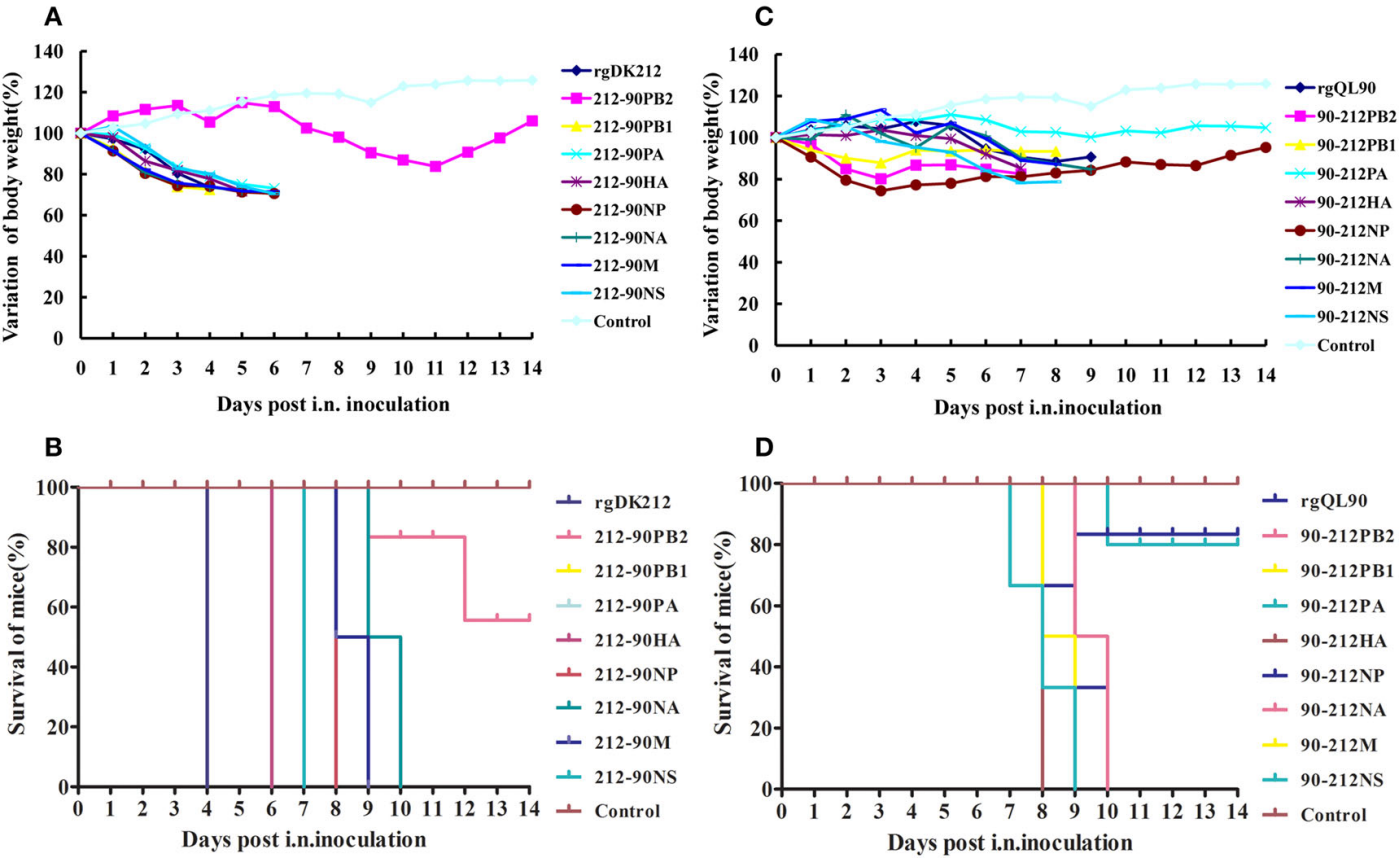
**Lethality and weight variation of BABL/c mice caused by rgDK212, rgQL90, and their chimeric viruses**. **(A,C)** Weight variation of BABL/c mouse during the 14 days post-inoculation. The mean weight variation of the mice infected with rgDK212, rgQL90 and their and chimeric viruses of which one gene was replaced with the corresponding that of QL90, at the doses of 10^6^ EID_50_. **(B,D)** Death patterns of the mice infected with rgDK212, rgQL90and their chimeric viruses, with the doses of 10^6^ EID_50_.

To detect the pathogenicity of a virus-bearing single segment swap, mice were intranasally infected with the corresponding virus at a dose of 10^6^ EID_50_. Virus 212-90PB2 only caused 16.15% body weight loss and caused 60% mice to die (Figures 2A,B). The 212-90PB2 was only detected in the lungs, and the titer was significantly lower than that of rgDK212 (Table 2). The MLD_50_ of 212-90PB2 virus was 1896-fold lower than that of DK212 (Table 3). The PB2 of DK212 was crucial to its high virulence in mice.

### The swap of PA–NA and M could slightly decrease the virulence of DK212 in mice

Besides PB2, the swap of PA–NA and M slightly attenuated the virus. 212-90PA, 212-90NA, and 212-90M caused 26.85–28.44% body weight loss and killed all mice by 8–10 DPI. Most viruses caused a systemic infection and then replicated. The mean titer was 1.08–5.75 log_10_EID_50_ and the MLD_50_ was between 2.045 and 2.375. The virulence of these viruses was attenuated 7.55–16.14 folds as compared to rgDK212 (Tables 2, 3). Notably, the 212-90M virus displayed no replication in the brain and lower replication in the kidneys and lungs (*p* < 0.05).

### The swap of PB1, NP and NS maintains the virulence of DK212 in mice

The chimeric viruses 212-90PB1, 212-90NP, and 212-90NS caused 27.46–29.43% body weight loss and killed all mice by 7–8 DPI. Similar to DK212, these three chimeric viruses replicated in four organs and the mean titer was 1.58–6.75 log_10_EID_50_. The replication titer of 212-90NS in the kidneys was higher than that of rgDK212 (*p* < 0.05). The MLD_50_ values of these three viruses were between 1.167 and 1.35, and maintained similar virulence to rgDK212.

### The swap of HA slightly increased the virulence of DK212 in mice

The chimeric virus 212-90HA caused 28.23% body weight loss and killed all mice at 6 DPI. Although the MLD_50_ of 212-90HA was 0.375, which is 6.19-fold higher than rgDK212, 212-90HA replicated in the brain, spleen and lung, Mean titer was 1.25–6.58 log_10_EID_50_ and the replication titer in the spleen was lower than rgDK212 (*p* < 0.05).

### The swap of PB2 obviously increased the virulence of QL90 in mice

The 90-212PB2 virus caused 17.53% body weight loss and killed all the mice at 8 DPI at a dose of 10^6^ EID_50_ (Figures 2C,D). Although 90-212PB2 had similar replication patterns as rgQL90, the virulence of 90-212PB2 was 58.88(10^1.77^)-fold higher than that of rgQL90 (Tables 2, 3).

### The NS gene of DK212 significantly enhanced pathogenicity of QL90 in mice

90-212NS viruses caused 21.32% body weight loss and killed all mice at 9 DPI at a dose of 10^6^ EID_50_. The swap of NS enabled the QL90 virus to replicate in the brain, kidneys and lung with a titer higher than rgQL90 (*p* < 0.05) (Table 2). In addition, the virulence of 90-212NS in mice was 133.35(10^2.125^)-fold higher than that of rgQL90 (Table 3).

### The PB1 gene of DK212 slightly increased pathogenicity of QL90 in mice

90-212PB1 caused 12.19% body weight loss and killed all mice at 9 DPI at a dose of 10^6^ EID_50_. Compared to rgQL90, 90-212PB1 showed no detected replication in the spleen and had a lower replication titer in the lungs with 4.08 log_10_EID_50_ (*p* < 0.05). However, the virulence of 90-212PB1 was 5.31-fold higher than that of rgQL90.

### The swap of HA, NP, NA, and M maintain the virulence of QL90 in mice

90-212HA, 90-212NA, and 90-212M caused 14.77–15.26% body weight loss and killed all mice at 8–10 DPI, at a dose of 10^6^ EID_50_. These three viruses showed replication in multiple organs with a titer of 1.08–6.17 log_10_EID_50_. The titer of 90-212HA in the kidneys was higher than that of rgQL90 (*p* < 0.05) (Table 2). The MLD_50_ of these three viruses were 4.167–4.276. The 90-212NP virus at most caused 25.64% body weight loss and killed 60% of mice (Figures 2C,D). 90-212NP only replicated in the spleen and lung. The MLD_50_ of 90-212NP was 4.445 (Table 3).

### The swap of PA significantly decreased the virulence of QL90 in mice

90-212PA had no obvious effect on body weight, and killed 20% of mice at a dose of 10^6^ EID_50_ (Figures 2C,D). 90-212PA replicated in the spleen, kidneys and lungs with a titer of 1.92–5.25 log_10_EID_50_ (Table 2). The MLD_50_ of 90-212PA was 6.25, 120.23(10^2.08^)-fold lower than that of rgQL90 (Table 3).

### The PB2 715Ser mainly decided the high virulence of DK212 in mice

**Figure 3:**
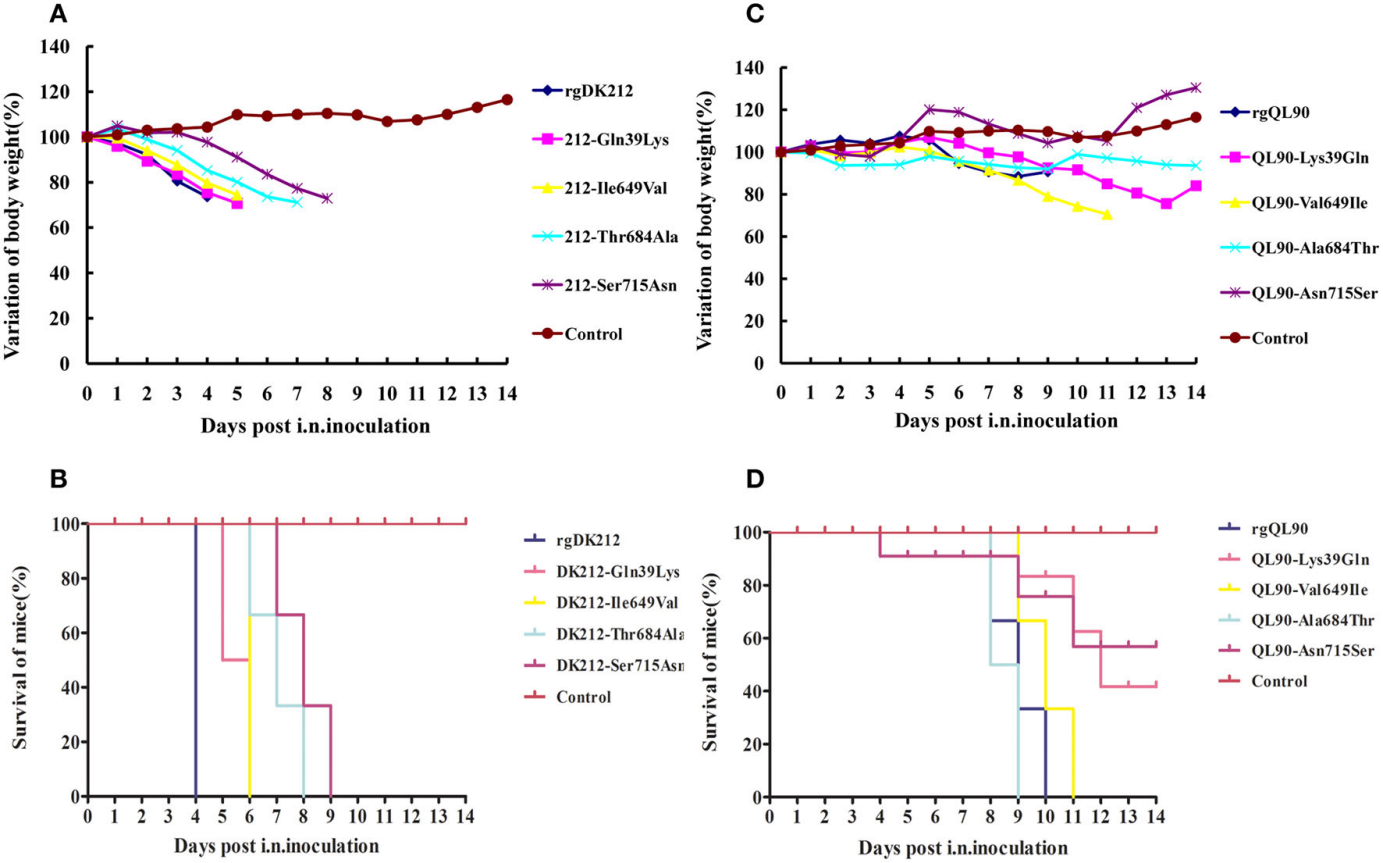
**Lethality and weight variation of BABL/c mice caused by rgDK212, rgQL90, and their four mutants**. **(A,C)** Weight variation of BABL/c mouse during the 14 days post-inoculation. The mean weight variation of the mice infected with rgDK212, rgQL90 and their single amino acid mutants, at the doses of 10^6^ EID_50_. **(B,D)** Death patterns of the mice infected with rgDK212, rgQL90 and mutants viruses of which single amino acids of PB2 gene was substitution, with the doses of 10^6^ EID_50_.

To further determine the PB2 differences between these two viruses in pathogenicity to mice, we selected four single sites to generate mutants with a single amino acid swap, and detected their pathogenicity in mice. The 212-Gln39Lys, 212-Ile649Val, 212-Thr684Ala, and 212-Ser715Asn viruses caused 25.57–29.24% body weight loss, and killed all the mice at 6–9 DPI. These four mutant strains could replicate in multiple organs with a titer of 1.08–7 log_10_EID_50_. The titer of 212-Thr684Ala in the kidneys was lower than that of rgDK212 (*p* < 0.05). The MLD_50_ of these four viruses were 2.464–4.6, and were 19.82–2710.19-fold lower than rgDK212. Notably, the Ser715Asn mutation sharply decreased the virulence of rgDK212 in mice (2710.19-fold) (Figures 3A,B, Tables 2, 3). These results indicated that 715Ser made a great contribution to the high pathogenicity of DK212 in mice.

### The single amino acid mutation decreased the virulence of QL90 in mice

The 90-Lys39Gln, 90-Val649Ile, 90-Ala684Thr and 90-Asn715Ser caused 2.12%–29.48% body weight loss, and killed 40–100% of mice. 90-Ala684Thr could replicate in the spleen and kidneys, but the other three mutants only replicated in the lungs with a titer of 3.58–5.75 log_10_EID_50_, at a dose of 10^6^ EID_50_. The results of MLD_50_ indicated the respective virulence of these four viruses were 2.19–38.02 folds lower than that of rgQL90 (Figures 3C,D, Tables 2, 3).

### The PB2 of DK212 was involved in high polymerase activity

**Figure 4:**
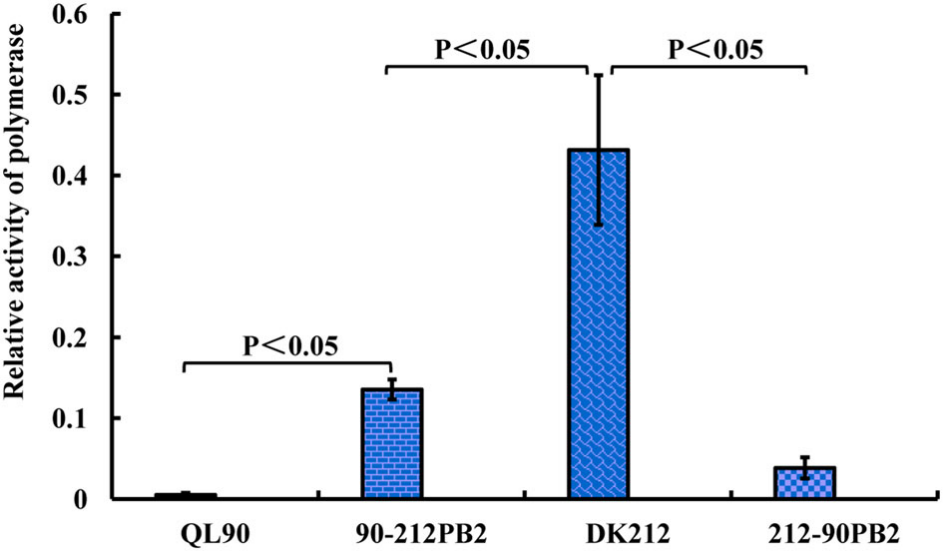
**Relative activity of polymerase complexes of QL90, 90-212PB2, DK212, and 212-90PB2**. The 293T cells were transfected with pHH-NP, pHH-PA, pHH-PB1, and pHH-PB2, along with the luciferase and renilla reporter gene plasmids. Transfected cells were cultured at 37 for 24 h, and luciferase and renilla production were determined. The relative activity of polymerase complexes was expressed as the production of luciferase dividing the production of renilla. The results were calculated as means ± standard deviations from three independent experiments, by using of SPASS (version 11.5).

To compare the PB2 role of these two viruses in polymerase complexes, polymerase activity assay was conducted. The activity of polymerase complexes of DK212 was 85-fold higher than QL90 in 293T cells at 37°C. After the swap of PB2 between these two viruses, 90-212PB2 polymerase complex activity was 26-fold higher than QL90 at 37°C. In contrast, the activity of 212-90PB2 complex was 11-fold lower than that of the DK212 complex (Figure 4). These results indicated that the PB2 gene was heavily involved in the discrepancy between the polymerase activity levels of these two viruses in human 293T cells at 37°C.

## Discussion

H5N1 highly pathogenic avian influenza virus could be transmitted to ferrets by aerosol droplets due to the acquisition of some mutations in HA and PB2 in lab experiments. This kind of novel virus may aggravate the threat to public health posed by H5N1 HPAIV. Therefore, studying the molecular mechanism of the pathogenicity of H5N1 virus in mammalian models is still emergent and necessary.

The PB2 protein plays an important role in pathogenicity. Mutation of Glu 627Lys or 701Asn in PB2 increases the pathogenesis of viruses in mammalian hosts (Li et al., 2005; de Jong et al., 2013) and transmissibility between mammalian models (Gabriel et al., 2008; Gao et al., 2009; Steel et al., 2009). In addition, mutation Gln591Lys of PB2 could compensate for the lack of PB2-627Lys and increase the virulence of an avian H5N1 influenza virus in mice (Yamada et al., 2010). In this study, the amino acids at positions 591, 627, and 701 in PB2 were Gln, Glu, and Asp for DK212 and QL90, but both of them still had high levels of pathogenicity in mice. The results support the contribution of the virus without 627Lys or 701Asn or Gln591Lys could be compensated for by other mutations at other sites of PB2. The virulence of DK212 showed 464-fold higher than that of QL90 in mice. The swap of PB2 sharply decreased the virulence of rgDK212 (1896-fold) and limited replication of rgDK212 to the lungs only and with lower titer in mice. In contrast, the swap of PB2 dramatically increased the virulence of rgQL90 in mice (60-fold). The results indicated PB2 was a key factor for determining differences in the virulence of DK212 and QL90 in mice.

The PB2 protein is also involved in polymerase activity and nuclear localization. PB2 acts as a “cap snatching” function in polymerase complex of influenza. The cap binding sites of PB2 locates in residues 32–483. The C-terminal of PB2 (residues 538–759) interacts with host proteins. Introduction of mutations in the PB2 gene, such as 339 Lys, 271Ala, 627Lys, 591Lys/Arg, 701Asn, and 714Arg enhance polymerase activity in mammalian cells (Gabriel et al., 2005; Bussey et al., 2010; Yamada et al., 2010; Liu et al., 2013). In this study, the different amino acids include Gln39Lys, near the domain of PB2 which binds to PB1; Thr339Lys, Arg340Lys, and Gly368Arg, locate in “cap snatching”; Ile649Val, Thr684Ala, and Ser715Asn, locate in the domain in which PB2 reacts with the host protein. Mutations were located in or near the regions in which proteins or protein/RNA interactions affected the activity of polymerase complex. Swapping the PB2 gene significantly enhanced the polymerase activity of QL90 (26-fold) and sharply reduced the activity polymerase complex of DK212 (11-fold) in human 293T cells at 37°C. These results indicated that PB2 of DK212 played an important role in maintaining high polymerase activity. The different properties of the PB2 in DK212 and QL90 might account for their differing replication ability in mice.

The NS1 protein is an important factor in virulence by antagonizing IFN-α/β production for avian influenza viruses in a mammalian model (Cheung et al., 2002; Quinlivan et al., 2005; Li et al., 2006). NS1 could reduce the production of IFN-α/β by binding to dsRNA or retinoic-acid inducible gene I (RIG-I) ligands to block activation of 2′-5′-oligoadenylatesynthetase (OAS) and protein kinase R (PKR). NS1 also could suppress IFN pre-mRNA processing and mRNA nuclear exports, by binding to the cellular pre-mRNA processing protein cleavage and polyadenylation specificity factor (CPSF30) (Hale et al., 2008; Zhang et al., 2013). The H5N1 virus with some mutation, such as Asp92Glu, Pro42Ser, Leu103Phe, and Ile106Met in NS1, could enhance the ability to inhibit the IFN-α/β production and increase viral replication, resulting in high pathogenicity in pigs or mice (Seo et al., 2002; Twu et al., 2007; Jiao et al., 2008). In addition, the C-terminus of the NS1, PDZ-ligand domain was involved with the virulence level (Jackson et al., 2008). There were 4 amino acid discrepancies in NS1 and NS2 between DK212 and QL90, respectively. Swapping the NS gene could dramatically enhance the pathogenicity of rgQL90 (133.35-fold) and enable rgQL90 replication in the brain. The swap of NS gene did not obviously affect the pathogenicity and replication of DK212 in mice. These findings indicated the differences in NS protein between DK212 and QL90 could possibly play a key role in determining virulence. In addition, the higher activity of the polymerase complex of DK212 could counteract the effects of swapping the NS gene and maintaining the virulence of DK212 in mice.

HA protein serves as binding and fusogenic function during influenza A virus infection. Amino acid substitutions in HA which affect the receptor binding preference may alter tissue tropism and change virulence in the host (Schrauwen et al., 2014). The multi-basic cleavage site was critical for the spread of the H5N1 virus to the mouse brain following intranasal infection (Hatta et al., 2001). In this study, despite DK212 and QL90 having the same multi-basic cleavage site, there were 7 amino acid discrepancies in HA. QL90 was not similar to DK212 and only replicated in the spleen and lungs. The swap of HA increased the virulence of rgDK212 19.72-fold but also made rgDK212 lose the ability to replicate in the kidneys. Meanwhile, the swap of HA enabled rgQL90 replication in the brain and kidneys. These results indicated that HA of DK212 was important for the virus in maintaining the multiple-tissue tropism.

Non-coding sequences at the vRNA segments were parts of the viral RNA packing signals, mutation in no-coding area would affect viral RNA synthesis (Muster et al., 1991; Zheng et al., 1996; Watanabe et al., 2003; Liang et al., 2005; Ng et al., 2008). In addition, the coding region located at the end of the neuraminidase segment played an important role in RNA segment incorporation into virion (Fujii et al., 2005). 22 nucleic acids displayed differences in the non-coding and coding regions of the NA gene between DK212 and QL90. The differences in these nucleic acids of NA affected viral RNA synthesis and the formulation of virion, and subsequently attenuated the pathogenesis of DK212 virus in mice.

There were 7 amino acids discrepancy in PB2 between DK212 and QL90, which included three discrepancies at PB2 Ile649Val, Thr684Ala, and Ser715Asn located in the nuclear localization domain or host protein binding domain (Brown, 2000), and one discrepancy at Gln39Lys PB2, near the PA binding area (residues 1–35) (He et al., 2008; Sugiyama et al., 2009). Their mutation may disturb nuclear localization and interrupt the host's antiviral pathway to change the viral replication. Therefore, we chose these four sites to generate the single amino acid mutant. The virulence of 212-Gln39Lys, 212-Ile649Val, 212-Thr684Ala, and 212-Ser715Asn showed as being 19.82–2710.19-fold lower than in rgDK212. It is noteworthy that the Ser715Asn mutation dramatically decreased the virulence of DK212 by almost 2710.19-fold. Similar to rgDK212, the single amino acid swap decreased the virulence of rgQL90 in mice (2.19–38.02-fold). Asn715 Ser mutation made QL90 only able to replicate in the lungs with a lower titer. The Ser714Arg single mutation introduced into SC35 did not increase the virulence of the virus in mice, but 714Arg was essential for SC35M in maintaining high pathogenicity. PB2 714Arg worked together with other mutations for the adaptation of SC35M (Gabriel et al., 2005). Likewise, the Ser715Asn single mutation sharply decreased the virulence of DK212, but the Asn715Ser single mutation did not increase the pathogenicity of QL90. 715Ser was essential for maintaining high pathogenicity of DK212, but not for QL90.

In light of the present study, PB2 of DK212, which dramatically affected the polymerase activity, mainly contributed to the difference in virulence of DK212 and QL90 in mice. In addition, 715Ser in the PB2 protein played an important role in maintaining high pathogenicity of DK212 to mice.

## Author contributions

Hailiang Sun conducted the experiments and analyzed the data and wrote the paper. Pengfei Cui participated in partial mouse experiment. Peirong Jiao, Yan Qi, Xiaokang Li, and Wenbao Qi provided technical instruction. Yafen Song and Chenggang Xu were involved in isolation of wild-type viruses. Ming Liao and Peirong Jiao desired the experiments and revised paper.

### Conflict of interest statement

The authors declare that the research was conducted in the absence of any commercial or financial relationships that could be construed as a potential conflict of interest.
