# Supplementary material for: PB2 segment promotes high-pathogenicity of H5N1 avian influenza viruses in mice
Source: Front Microbiol. 2015 Feb 10;6:73. doi: 10.3389/fmicb.2015.00073 (PMC4322641; doi:10.3389/fmicb.2015.00073)
Supplement: Supplementary file 1 [file DataSheet1.DOCX]

**Supplementary table 1. Primers for amplification of the full-length cDNAs of DK212 virus**

| Name of primer | Sequence of primers |
| --- | --- |
| pDL-212PB2-F | 5'GTCTC*CGTCTC*AGGGGAGCAAAAGCAGGTCAAATATATTCAATA3' |
| pDL-212PB2-R | 5'GTCTC*CGTCTC*ATATTAGTAGAAACAAGGTCGTTTTTAAACAAT3' |
| pDL-212PB1-F | 5'GTCTC*CGTCTC*AGGGGAGCAAAAGCAGGCAAACCATTTGAA3' |
| pDL-212PB1-R | 5'GTCTC*CGTCTC*ATATTAGTAGAAACAAGGCATTTTTTCATG3' |
| pDL-212PA-F | 5'GTCTC*CGTCTC*AGGGGAGCAAAAGCAGGTACTGATCCAAAATGG3' |
| pDL-212PA-R | 5'GTCTC*CGTCTC*ATATTAGTAGAAACAAGGTACTTTTTTGGACAG3' |
| pDL-212HA-F | 5'GTCTC*CGTCTC*AGGGGAGCGAAAGCAGGGGTATAATCTGTCAAAATGGAG3' |
| pDL-212HA-R | 5'GTCTC*CGTCTC*ATATTAGTAGAAACAAGGGTGTTTTTAACTACAATCTGA3' |
| pDL-212NP-F | 5'GTCTC*CGTCTC*AGGGGAGCAAAAGCAGGGTAGATAATCACTCACCGA3' |
| pDL-212NP-R | 5'GTCTC*CGTCTC*ATATTAGTAGAAACAAGGGTATTTTTCTTTAATTGT3' |
| pDL-212NA-F | 5'GTCTC*CGTCTC*AGGGGAGCAAAAGCAGGAGTTTAAAAT3' |
| pDL-212NA-R | 5'GTCTC*CGTCTC*ATATTAGTAGAAACAAGGAGTTTTTTG3' |
| pDL-212M-F | 5'GTCTC*CGTCTC*AGGGGAGCAAAAGCAGGTAGATGTTGAAAG3' |
| pDL-212M-R | 5'GTCTC*CGTCTC*ATATTAGTAGAAACAAGGTAGTTTTTTACT3' |
| pDL-212NS-F | 5'GTCTC*CGTCTC*AGGGGAGCAAAAGCAGGGTGACAAAAACAT3' |
| pDL-212NS-R | 5'GTCTC*CGTCTC*ATATTATTAGAAACAAGGGTGTTTTTTATC3' |

Note: *CGTCTC* underlined was the recognition site of restriction enzyme *Esp*3I. F represented the forward primers, and R represented backward primers.

**Supplementary table 2. Primers for amplification of the full-length cDNAs of QL90 viruses**

| Name of primer | Sequence of primers |
| --- | --- |
| pDL-90PB2-F | 5’ GTCTC*CGTCTC*AGGGGAGCAAAAGCAGGTCAAATATATTCAATAT3’ |
| pDL-90PB2-R | 5’GTCTC*CGTCTC*AGGGGAGTAGAAACAAGGTCGTTTTTAAACAACT3’ |
| pDL-90PB1-F | 5’ GTCTC*CGTCTC*AGGGGAGCAAAAGCAGGCAAACCATTTGAATGG3’ |
| pDL-90PB1-R | 5’ GTCTC*CGTCTC*ATATTAGTAGAAACAAGGCATTTTTTCACGAAGG3’ |
| pDL-90PA-F | 5'GTCTC*CGTCTC*AGGGGAGCAAAAGCAGGTACTGATCCAAAATGG3' |
| pDL-90PA-R | 5'GTCTC*CGTCTC*ATATTAGTAGAAACAAGGTACTTTTTTGGACAG3' |
| pDL-90HA-F | 5’GTCTC*CGTCTC*AGGGGAGCAAAAGCAGGGGTTCAATCTGTCAAAAT3’ |
| pDL-90HA-R | 5’GTCTC*CGTCTC*ACTAAACCAGAGATTGGTACCAAAAATAG3’ |
| PDL-90NP-F | 5’GTCTC*GCTCTTC*AGGGGAGCAAAAGCAGGGTAGATAATCACTCACCG3’ |
| PDL-90NP-R | 5’ GTCTC*GCTCTTC*ATATTAGTAGAAACAAGGGTATTTTTCTTTAATT3’ |
| pDL-90NA-F | 5’GTCTC*CGTCTC*AGGGGAGCAAAAGCAGGAGTTCAAAATGAATCC3’ |
| pDL-90NA-R | 5’GTCTC*CGTCTC*AGGGGAGTAGAAACAAGGAGTTTTTTGAACAAACT3’ |
| pDL-90M-F | 5’GTCTC*CGTCTC*AGGGGAGCAAAAGCAGGTAGATGTTGAAAG3' |
| pDL-90M-R | 5’GTCTC*CGTCTC*ATATTAGTAGAAACAAGGTAGTTTTTTACT3' |
| pDL-90NS-F | 5’GTCTC*CGTCTC*AGGGGAGCAAAAGCAGGGTGATAAAAACATAATGG3’ |
| pDL-90NS-R | 5’GTCTC*CGTCTC*ATATTAGTAGAAACAAGGGTGTTTTTTATCATTAAAT3’ |

Note: *CGTCTC* underlined was the recognition site of restriction enzyme *Esp*3I. F represented the forward primers, and R represented backward primers.

**Supplementary table 3. Primers for single amino acid mutation in PB2 gene of DK212 and QL90**

| Name of primer | Sequence of primers |
| --- | --- |
| DK212-39F | AATACACATCAGGAAGAAAAGAGAAGAATCCTGCTC |
| DK212-39R | GAGCAGGATTCTTCTCTTTTCTTCCTGATGTGTATT |
| DK212-649F | GAGGCTCAGGAATGAGAATACTCGTAAGGGGCAATT |
| DK212-649R | AATTGCCCCTTACGAGTATTCTCATTCCTGAGCCTC |
| DK212-684F | GAGGATCCAGATGAGGGGACAGCCGGAGTGGAATCT |
| DK212-684R | AGATTCCACTCCGGCTGTCCCCTCATCTGGATCCTC |
| DK212-715F | TCAATGAACTGAGCAATCTTGCAAAAGGGGAGAAA |
| DK212-715R | TTTCTCCCCTTTTGCAAGATTGCTCAGTTCATTGA |
| QL90-39F | AATACACATCAGGAAGACAAGAGAAGAACCCTGCTC |
| QL90-39R | GAGCAGGGTTCTTCTCTTGTCTTCCTGATGTGTATT |
| QL90-649F | GGCTCAGGAATGAGAATACTCATAAGGGGCAATT |
| QL90-649R | AATTGCCCCTTATGAGTATTCTCATTCCTGAGCC |
| QL90-684F | CAGATGAGGGGACAACCGGAGTGGAATCT |
| QL90-684R | AGATTCCACTCCGGTTGTCCCCTCATCTG |
| QL90-715F | TCAATGAACTGAGCAGTCTTGCGAAAGGGGAG |
| QL90-715R | CTCCCCTTTCGCAAGACTGCTCAGTTCATTGA |

Note: The nucleotide underlined was the mutation site, F represented the forward primers, and R represented backward primers.
